# Measurement of human abdominal and femoral intravascular adipose tissue blood flow using percutaneous Doppler ultrasound

**DOI:** 10.1080/21623945.2021.1888471

**Published:** 2021-02-16

**Authors:** Ioannis G. Lempesis, Gijs H. Goossens, Konstantinos N. Manolopoulos

**Affiliations:** a Institute of Metabolism and Systems Research (IMSR), College of Medical and Dental Sciences, University of Birmingham, Birmingham, UK; b Centre for Endocrinology, Diabetes and Metabolism, Birmingham Health Partners, Birmingham, UK; c Department of Human Biology, NUTRIM School of Nutrition and Translational Research in Metabolism, Maastricht University Medical Centre+, Maastricht, The Netherlands

**Keywords:** Adipose tissue, fat depots, blood flow, Doppler ultrasound, human physiology

## Abstract

Adipose tissue blood flow (ATBF) is an important determinant of adipose tissue (AT) function. ^133^Xenon wash-out technique is considered the gold-standard for human ATBF measurements. However, decreasing ^133^Xenon clinical use and costly production and preservation, make alternative (non-invasive) methods necessary. Here, we explored percutaneous Doppler ultrasound as a proxy method to quantify intravascular subcutaneous abdominal and femoral ATBF in humans (*n*= 17). Both fasting ATBF and the postprandial increase in ATBF were significantly higher in abdominal compared to femoral AT. Although anatomical variations in vein location and depot thickness may impact feasibility, we demonstrate that Doppler ultrasound detects the expected depot-differences and postprandial increase in ATBF in healthy individuals. This method warrants further investigation in other populations and metabolic conditions.

## Introduction

Tissue-specific regulation of blood flow is needed to meet local physiological demands and to allow proper functioning of organs. Adipose tissue blood flow (ATBF) is an important determinant of adipose tissue (AT) function, as it delivers nutrients and oxygen to AT, exerts a key role in fatty acid trafficking, and distributes adipokines and metabolites into the circulation [1–3]. Under fasting conditions, nitric oxide, adrenergic regulation and the renin-angiotensin system determine ATBF [4,5], while the increase in ATBF following ingestion of a glucose drink or mixed meal is mainly under beta-adrenergic control [1]. Many studies have demonstrated that both fasting and the postprandial enhancement of ATBF are impaired in obesity and insulin-resistant conditions [6–8], thereby contributing to the metabolic perturbations in insulin-resistant individuals with obesity [1,3,7–10]. In line with an important role of ATBF in metabolic regulation, several, but not all, studies have demonstrated a lower ATBF in lower-body compared to upper-body AT [11–13].

The gold-standard method for measuring ATBF is the ^133^Xenon (^133^Xe) wash-out technique, first described by Larsen and colleagues in 1966 [14]. The rate of disappearance of the lipid-soluble radioactive isotope ^133^Xe, of which a small volume is administered in an area of subcutaneous AT, is used as an indicator of ATBF [10]. Alternative methods to assess subcutaneous ATBF include measurement of ethanol wash-out using microdialysis, laser Doppler flowmetry, and contrast-enhanced ultrasound [10,15–18]. The current reduced availability of ^133^Xe due to its costly production and declining clinical use, as well as the invasive nature of some of the other techniques implies the need for an alternative, non-invasive method for the measurement of ATBF that can be used in physiological human *in vivo* studies. The aim of the present study, therefore, was to explore intravascular Doppler ultrasound as a proxy method for measuring ATBF in humans, by establishing technical feasibility, reproducibility, and sensitivity of the method to detect ATBF changes in response to an oral glucose drink.

## Research design and methods

### Study design

Seventeen healthy individuals with no known medical conditions who were non-smokers and not taking any medication participated in the present study. Participants arrived at the Clinical Research Facility in the morning around 08:30 a.m. after an overnight fast (at least 10 h fasting). They were advised to abstain from coffee, tea and alcohol, and sports or intense physical activity one day prior to the measurements, but to keep on their usual activities. Following 30 min of rest, two ATBF measurements in abdominal and femoral AT, separated by 5 min, were acquired under fasted conditions. Next, subjects were asked to ingest 75 g of glucose in the form of a pre-made drink (113 ml of Polycal Liquid, Nutricia Ltd, Trowbridge, Wiltshire, UK). Postprandial ATBF responses in abdominal and femoral AT were determined for 120 min at 10 min intervals. Measurements were taken by a single operator and, depending on feasibility, repeated up to three times at each time-point for both AT depots. Femoral ATBF was measured in 11 individuals due to a near-parallel to the skin anatomical course of femoral AT veins resulting in poor Doppler signal. The study was approved by the University of Birmingham Ethics committee and the UK Health Research Authority. All participants provided written informed consent before taking part in the study procedures.

### Doppler ultrasound technique

Vessels that specifically drain abdominal subcutaneous AT are branches of the superficial epigastric vein (*V. epigastrica superficialis*), that are located above the inguinal ligament, as determined by using the anterior superior iliac spine and the projected pubic symphysis as reference points [19]. In the present study, the subcutaneous AT areas lateral of the umbilicus and between the lower end of the rib cage and the inguinal ligament were scanned on each side in order to identify suitable veins for ATBF measurements in the abdominal subcutaneous AT depot. In the femoral depot, the great saphenous vein and its branches drain mostly femoral subcutaneous AT [20]. Suitable femoral veins for ATBF measurements were identified by scanning the inner aspect of the thigh, approximately half-way between the groin and the knee. A Philips CX50 ultrasound system (Philips Ultrasound, 22,100 Bothell-Everett Highway, Bothell, WA 98,021–8431, USA) with two different transducers was used for the measurements: Depending on the anatomical location, course and size of the identified veins, a L15-7io broadband compact linear array transducer (Frequency range: 15–7 MHz) and a L12-3 (Frequency range: 12–3 MHz) were used.

**Figure 1.**
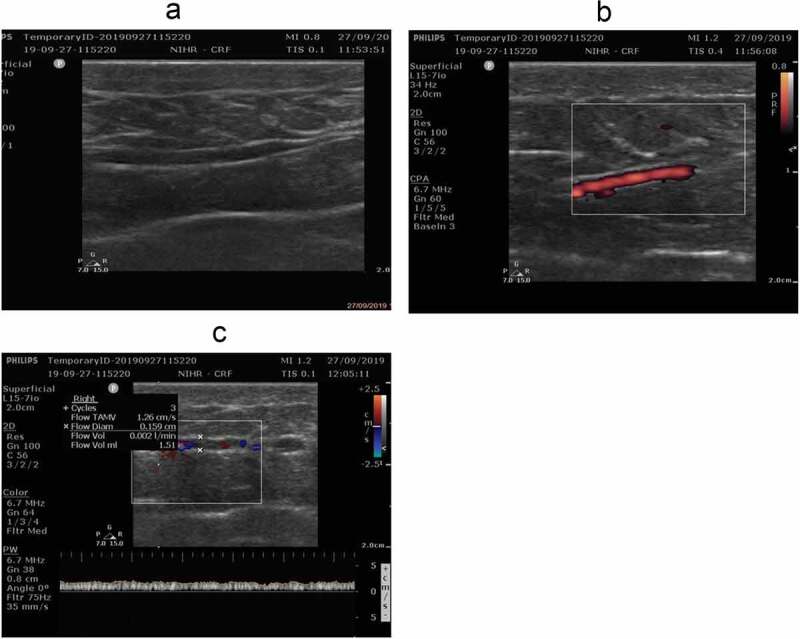
Representative images from the workflow of obtaining an ATBF measurement using Doppler ultrasound. Identification of a suitable subcutaneous adipose tissue vein (a), confirmation of flow signal in colour power angio (PCA) mode (b), followed by measurement of vessel diameter and calculation of ATBF (c)

The sequence of measurement involved the following steps. One or two suitable subcutaneous veins draining the respective AT depot were identified using a live greyscale imaging mode (2D Mode; Figure 1(a)), and their location was marked on the skin with a suitable marker for easy identification for measurements later during the study. The insonation angle was checked visually between two projected lines, one corresponding to the skin level and the other running horizontally across the vessel lumen, aiming for it to be less than 70 degrees to ensure an adequate Doppler signal. Flow within the selected vein was visualized using colour or colour power angio (CPA) mode (Figure 1(b)), before switching to pulsed waved (PW) Doppler to obtain the measurement. The quality of the Doppler signal was optimized using proprietary functions of the ultrasound system (iSCAN Intelligent Optimization on the system used in this study). Following the conduction of the PW Doppler measurement, the live image was frozen and flow volume calculations were performed using the ultrasound system’s internal algorithms to obtain a time-averaged flow. Importantly, this included measurement of the vessel diameter (Figure 1(c)). During the PW Doppler measurement, great care was taken to apply the lowest possible pressure to the skin with the ultrasound transducer to avoid compression of the vessel, which could affect ATBF.

### Statistical analyses

For each time point, mean ATBF was determined from the available repeated measurements. Fasting ATBF was calculated as the mean of time points t = −5- and 0-min. Peak ATBF was defined as the highest value recorded during the measurements’ timeframe. Time-averaged area under the curve (iAUC/min) was calculated following the trapezoid rule. Since parameters were not normally distributed (based on Shapiro-Wilk test), the Wilcoxon-Signed Rank test was used to compare fasting and postprandial ATBF within the same AT depot, and to compare ATBF between abdominal and femoral AT. SPSS version 25 and GraphPad Prism version 8 were used to perform statistics, and p < 0.05 was considered as statistically significant.

## Results

**Figure 2.**
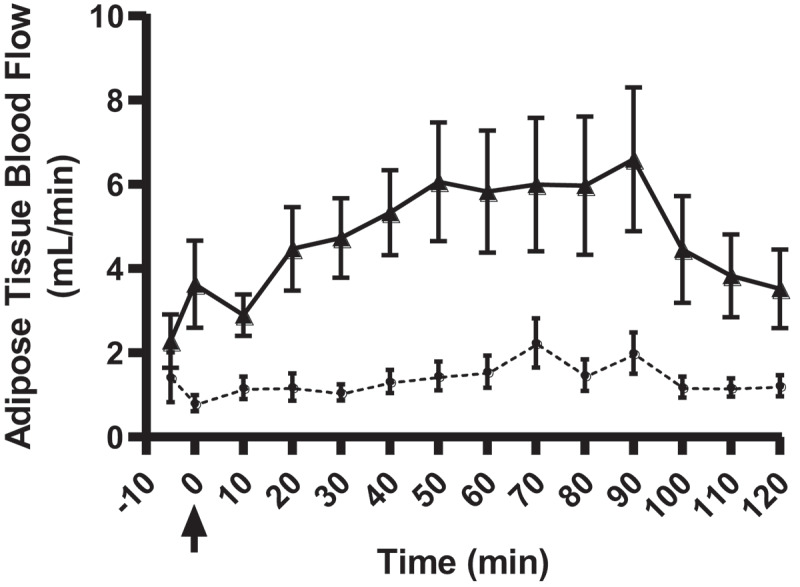
Abdominal (solid line with triangles, *n*= 17) and femoral (dashed line with circles, *n*= 11) adipose tissue blood flow measurements with Doppler ultrasound. A standardized 75 g glucose drink was given at time 0 min (black arrow). ATBF follows the expected postprandial increase for the abdominal adipose tissue depot, peaking at 6.9 ± 1.7 mL/min (t = 90 min post-glucose ingestion) (p = 0.002 vs. fasting ATBF, *n*= 17). The femoral AT depot showed a less pronounced increase in ATBF to 2.2 ± 0.6 mL/min (peak value at t = 70 min post-glucose ingestion) (p = 0.047 vs. fasting ATBF, *n*= 11). The postprandial enhancement (iAUC) in abdominal ATBF was more pronounced than in femoral AT (p = 0.033, *n*= 11)

The participant characteristics are shown in Table 1. Briefly, seventeen individuals (12 females, 5 males) with BMI range 19.8–33.8 kg/m^2^ and age range 24–58 years were included in the present study. Of note, only one study participant had obesity (BMI, 33.8 kg/m^2^) with the BMI range of all other participants being 19.8–24.8 kg/m^2^. Fasting abdominal ATBF was 2.9 ± 0.8 mL/min, which increased to peak levels at 6.9 ± 1.7 mL/min at t = 90 min after glucose ingestion (p = 0.002 vs. fasting ATBF) (Figure 2). Femoral ATBF increased from 1.1 ± 0.3 mL/min under fasting conditions to peak ATBF values of 2.2 ± 0.6 mL/min at t = 70 min after glucose ingestion (p = 0.047 vs. fasting ATBF).

**Table 1.** Participants’ characteristics

Sex	5 Males/12 Females
Age (years)	37 (24–58)
Height (m)	1.73 (1.58–1.88)
Weight (kg)	69.1 (49.5–111.4)
BMI (kg/m^2^)	22.9 (19.8–33.8)
Waist circumference (cm)	83.4 (68–119)
Hip circumference (cm)	99.4 (89.5–117)
Waist/Hip ratio	0.84 (0.69–1.01)

BMI: Body Mass Index

Fasting ATBF was higher in abdominal compared to femoral AT (2.9 vs. 1.1 ml/min, respectively, p = 0.033, *n*= 11 paired measurements). Furthermore, the postprandial increase in ATBF (iAUC/min) was significantly higher in abdominal than femoral AT (2.0 vs. 0.3 ml/min, respectively, p = 0.033, *n*= 11 paired measurements). The iAUCs for both abdominal and femoral ATBF were not significantly correlated with BMI (abdominal: r = −0.018, p = 0.945; femoral: r = −0.388, p = 0.237) or waist/hip ratio (abdominal: r = 0.076, p = 0.773; femoral: r = 0.582, p = 0.065). When examining the individual participants where a femoral ATBF measurement was not possible, there were no notable predictors of poor signal apart from the anatomical considerations outlined above. The coefficient of variation of repeated measurements in abdominal AT was 33 ± 6% for fasting ATBF and 20 ± 5% for peak ATBF. For femoral AT, the coefficient of variation of repeated measurements was 39 ± 7% for fasting ATBF and 51 ± 11% for peak ATBF.

## Discussion

The present study demonstrated that measurement of abdominal and femoral intravascular ATBF with percutaneous Doppler ultrasound is technically feasible. This non-invasive method is able to detect the expected increase in blood flow following oral glucose ingestion in both abdominal and femoral AT in healthy individuals [1–3]. Furthermore, we found that abdominal ATBF was significantly higher than femoral ATBF under fasting conditions. Moreover, the postprandial increase in abdominal subcutaneous ATBF was significantly higher than the ATBF increase in femoral AT. These findings are in agreement with previous studies, in which ATBF was quantified using the gold-standard ^133^Xe wash-out technique [1,11]. As expected, we found that there is large inter- and intra-individual variability in ATBF, which is commonly observed with ATBF [8] measurements, even when performed using the ^133^Xe wash-out technique. However, intra-individual coefficients of variation with intravascular Doppler ultrasound seem larger than found with ^133^Xe wash-out [8].

Important limitations of the method employed in the present study are those inherent to using ultrasound and relate to the quality of the ultrasound image that can be obtained in individual participants. Appropriate operator training paying special attention to the identification of suitable veins that allow acquisition of a high-quality PW Doppler signal is of paramount importance. Low blood flow in small AT veins may further hamper the accuracy of ATBF measurement. These issues become especially noticeable when assessing ATBF in (very) lean participants due to thin subcutaneous fat layers and smaller blood vessels. While the anatomical properties of AT veins, especially in the femoral AT depot, may limit the ability of obtaining a good Doppler signal in every participant of a given study, it is important to note that other available methods also have intrinsic limitations such as exposure to radiation (i.e. PET-tracers, ^133^Xe wash-out), thereby restricting their applicability. This also holds true for repeated measurements during a clinical study, which is often not possible with PET scans, for example. Although intravascular Doppler ultrasound may be an alternative method that could be applied to calculate metabolite fluxes across AT in physiological *in vivo* studies in humans, it is important to note that Doppler ultrasound provides data on intravascular blood flow in relatively large AT veins. In contrast, the ^133^Xe wash-out technique on which the original calculations of metabolic fluxes were based provides ATBF values at the capillary level. Due to the global production stop of medical ^133^Xe it was not possible to validate the present Doppler measurements against the gold-standard ^133^Xe wash-out technique. A further limitation of our study is that we did not obtain information on the actual volume of the AT depot measured, e.g. through magnetic resonance imaging or dual x-ray absorptiometry, which could allow for the modelling of whole-depot blood flow values. Finally, we did not measure postprandial glucose and insulin concentrations in this study, although we would not expect to find any different postprandial glucose and insulin responses to previously published data given that all our participants were healthy volunteers [7].

To summarize, the present study demonstrates that AT Doppler ultrasound is a non-invasive method for measuring abdominal and femoral ATBF, that is sensitive enough to detect differences between AT depots as well as the expected postprandial increase in ATBF in healthy individuals. Future studies should explore the feasibility and sensitivity of this method in determining ATBF in other populations, for example individuals with obesity and patients with type 2 diabetes, establish inter-operator variability, and assess the usefulness of the method in combination with volumetric AT measurements as a basis of mathematical models allowing calculation of substrate fluxes across different AT depots.
